# Supplementary material for: HIVprotI: an integrated web based platform for prediction and design of HIV proteins inhibitors
Source: J Cheminform. 2018 Mar 9;10:12. doi: 10.1186/s13321-018-0266-y (PMC5845081; doi:10.1186/s13321-018-0266-y)
Supplement: Supplementary file 2 — Additional file 2. Source code of HIVProtI web server. [file 13321_2018_266_MOESM2_ESM.zip › HIVprotI_Source-code/hivprotI/weka-3-6-6/doc/weka/core/WeightedInstancesHandler.html]

WeightedInstancesHandler


---


|  |  |  |
| --- | --- | --- |
| **Overview** \| **Package** \| **Class** \| **Tree** \| **Deprecated** \| **Index** \| **Help** \| **Weka's home** | |  |
| **PREV CLASS**   **NEXT CLASS** | **FRAMES**    **NO FRAMES**     **All Classes** |  |
| SUMMARY: NESTED \| FIELD \| CONSTR \| METHOD | DETAIL: FIELD \| CONSTR \| METHOD |  |


---


## weka.core Interface WeightedInstancesHandler

**All Known Implementing Classes:**: AdaBoostM1, AdditiveRegression, ADTree, AODE, AODEsr, AttributeSelectedClassifier, Bagging, BayesNet, BayesNetGenerator, BIFReader, ComplementNaiveBayes, ConjunctiveRule, DecisionStump, DecisionTable, Discretize, Discretize, DMNBtext, DTNB, EditableBayesNet, EM, FTInnerNode, FTLeavesNode, FTNode, FTtree, IBk, IsotonicRegression, J48, J48graft, JRip, LinearRegression, LMTNode, Logistic, LogisticBase, LogitBoost, LWL, MakeDensityBasedClusterer, MIOptimalBall, MISMO, MultiBoostAB, MultilayerPerceptron, NaiveBayes, NaiveBayesMultinomial, NaiveBayesMultinomialUpdateable, NaiveBayesUpdateable, NBTree, PaceRegression, PART, PKIDiscretize, RandomCommittee, RandomForest, RandomSubSpace, RandomTree, REPTree, Ridor, RotationForest, Rule, SimpleKMeans, SimpleLinearRegression, SimpleLogistic, SMO, SMOreg, VFI, ZeroR

---

``` public interface WeightedInstancesHandler ```

Interface to something that makes use of the information provided
by instance weights.

**Version:**
:   $Revision: 1.5 $

**Author:**
:   Eibe Frank (eibe@cs.waikato.ac.nz)

---

---


|  |  |  |
| --- | --- | --- |
| **Overview** \| **Package** \| **Class** \| **Tree** \| **Deprecated** \| **Index** \| **Help** \| **Weka's home** | |  |
| **PREV CLASS**   **NEXT CLASS** | **FRAMES**    **NO FRAMES**     **All Classes** |  |
| SUMMARY: NESTED \| FIELD \| CONSTR \| METHOD | DETAIL: FIELD \| CONSTR \| METHOD |  |


---
